# Chemical Composition and in Vitro Antimicrobial, Cytotoxic, and Central Nervous System Activities of the Essential Oils of *Citrus medica* L. cv. ‘Liscia’ and *C. medica* cv. ‘Rugosa’ Cultivated in Southern Italy

**DOI:** 10.3390/molecules21091244

**Published:** 2016-09-18

**Authors:** Luigi Aliberti, Lucia Caputo, Vincenzo De Feo, Laura De Martino, Filomena Nazzaro, Lucéia Fátima Souza

**Affiliations:** 1 Department of Pharmacy, University of Salerno, Via Giovanni Paolo II, 132, 84084 Fisciano (Salerno), Italy; luigialiberti83@libero.it (L.A.); lcaputo@unisa.it (L.C.); ldemartino@unisa.it (L.D.M.); luceia.souza@ufrgs.br (L.F.S.); 2 Istituto di Scienze dell’Alimentazione, Consiglio Nazionale delle Ricerche (ISA-CNR), Via Roma 64, 83100 Avellino, Italy; mena@isa.cnr.it; 3 Post-Doctoral by National Counsel of Technological and Scientific Development (CNPq/Brazil), 70000-000 Brasília, Brazil

**Keywords:** *Citrus medica*, essential oil, antimicrobial activity, SH-SY5Y cells, adenylate cyclase

## Abstract

*Citrus medica* cv. ‘liscia’ and *C. medica* cv. ‘rugosa’ are two taxa of citron, belonging to the biodiversity of South Italy, in particular of Amalfi Coast, in the Campania region. The chemical composition of the essential oils (EOs) from fruit peels of both *C. medica* cultivars was studied by gas chromatography (GC) and gas chromatography-mass spectrometry (GC-MS) analyses. In all, 100 compounds were identified, 82 for *C. medica* cv. ‘liscia’, accounting for 91.4% of the total oil, and 88 for *C. medica* cv. ‘rugosa’, accounting for 92.0% of the total oil. Monoterpene hydrocarbons are the main constituents in both oils of *C. medica* cv. ‘liscia’ (79.1%) and *C. medica* cv. ‘rugosa’ (80.2%). In both oils, limonene (67.2%–62.8%) and camphene (8.5%–10.9%) are the main constituents. The antimicrobial activity of the EOs was assayed against some bacterial strains: *Bacillus cereus* (DSM 4313), *Bacillus cereus* (DSM 4384), *Staphylococcus aureus* (DSM 25693), *Pseudomonas aeruginosa* (ATCC 50071), and *Escherichia coli* (DSM 8579). Low concentrations of *C. medica* cv. ‘rugosa’ EO showed an inhibitory effect on *P. aeruginosa* and higher concentrations inhibited more *B. cereus* (4384) and *E. coli* than *S. aureus*. The cytotoxicity of the EO was evaluated against SH-SY5Y cell line. The influence of the EO on the expression of adenylate cyclase 1 (ADCY1) was also studied. The antimicrobial activity registered confirm their traditional uses as food preserving agents and led us to hypothesize the possible use of these oils as antimicrobials. The alterations in ADCY1 expression suggested a role for limonene in effects on the central nervous system.

## 1. Introduction

Citron, native to Southeast Asia, was imported to the Mediterranean around 300 B.C. Probably, it arrived in Italy through the Hebrews who introduced the cultivation of citron on the Calabrian coasts, Amalfi Coast, and Garda Lake [1,2].

Two local cultivars of *Citrus medica* L. are grown on the Amalfi Coast: *C. medica* cv. ‘liscia’, known by the vernacular name of ‘cedro’, and *C. medica* cv. ‘rugosa’, known as ‘ponsino’. These two cultivars contributed to the agricultural biodiversity of this area, as well as other *Citrus* species. However, their diffusion is decreasing, due to the technical difficulties for their cultivation and to the competition of lemon cultivations. The taxonomy of *Citrus* species is complex. In fact, recent genetic analyses have shown that only three species belong to the genus *Citrus*: *C. maxima* (Burm.) Merr., *C. medica* L., and *C. reticulata* Blanco [3]. Moreover, the *Citrus* species are able to crossbreed, producing fruits with a wide range of morphological and organoleptic characteristics.

Today, the fruits of both cultivars are used locally only for fresh alimentary consumption. In past times, both citrons have also been employed in traditional medicine as an anti-infective, an anti-inflammatory, and to treat digestive disorders.

These traditional uses agree with the folkloric uses of citron. In fact, fruits and leaves are used in different countries in the treatment of allergic inflammation, for treating colds, as a decongestant, an expectorant, and a carminative or, in the case of pathologies of the intestinal tract and rectum, as well as a stomachic, an antispasmodic, a diuretic and a digestive [3,4]. The citron essential oils (EOs) are used for flavoring, for perfuming, in fruit beverages, in soft drinks, in cosmetics, and in household products [4].

Different studies reported evidence that *Citrus* consumption is associated with a reduced cancer incidence [5]. Menichini and coworkers [6] reported the chemical profile and the photo-induced cytotoxic activity of *Citrus bergamia* Risso and Poit. and *Citrus medica* cv. ‘Diamante’. Both oils exhibited a selective inhibition of the A375 tumoral cell line. Russo and coworkers [7] studied the cytotoxic effect of the Bergamot EO on SH-SY5Y neuroblastoma cells and its components, limonene and linalyl acetate, were able to induce cell death.

Moreover, some EOs and their components are known for their activity on the central nervous system, interacting with *N*-methyl-d-aspartate (NMDA) receptor complex [8,9], and showing an inhibitory effect of the acetylcholine release and on the channel open time in the mouse neuromuscular junction [10].

The aims of this paper were to study the chemical composition of the EOs obtained from the peel of the fruits of the two cultivars grown in Amalfi Coast, to evaluate their potential antimicrobial activity, their cytotoxicity and the possible effects on central nervous system. 

## 2. Results

### 2.1. Essential Oil Yield and Composition

Hydrodistillation of the peel from fruits of *C. medica* cv. ‘liscia’ and *C. medica* cv. ‘rugosa’ gave yellow EOs characterized by a typical citrusy and floral odor, with yields of 0.9% and 0.75%, respectively. Table 1 shows the chemical composition of the two oils; the compounds are listed according to their elution order on an HP-5 MS capillary column. All 100 compounds were identified, 82 for *C. medica* cv. ‘liscia’, accounting for 91.4% of the total oil, and 88 for *C. medica* cv. ‘rugosa’ accounting for 92.0% of the total oil. Monoterpene hydrocarbons are the main constituents in both oils, 79.1% for cv. ‘liscia’ and 80.2% for cv. ‘rugosa’. In both oils, limonene (67.2%–62.8%), camphene (8.5%–10.9%), and β-pinene (1.4%–1.7%) were other main components. In the oil from *C. medica* cv. ‘liscia’ other components in a lesser amount are geranyl acetate (0.9%), and α-*trans*-bergamotene (0.5%); in the oil from cv. ‘rugosa’ geraniol (0.7%), geranial (0.7%), neral (0.5%), isopulegol (0.7%), and α-bisabolol (0.5%) are present.

### 2.2. Antibacterial Activity

**Figure 1:**
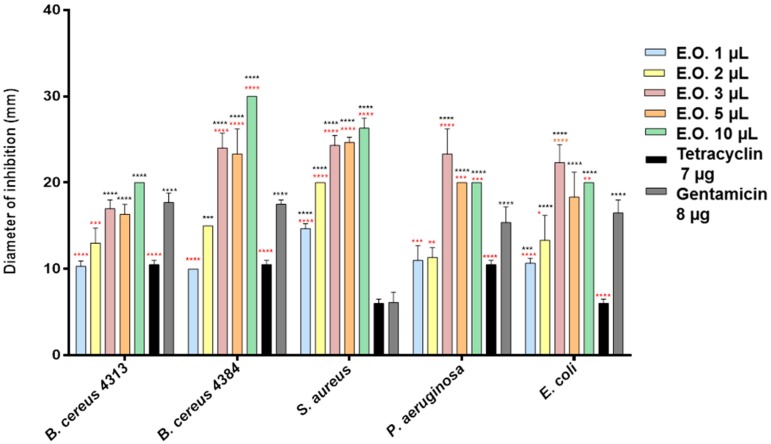
Antibacterial activity of essential oil (EO) of *Citrus medica* cv. ‘liscia’ and of the reference compounds, gentamicin and tetracycline. Results are the mean of three experiments. Dunnett’s test vs. gentamicin 8 μg: **** *p* < 0.0001; *** *p* < 0.001; ** *p* < 0.01; * *p* < 0.05; Dunnett’s test vs. tetracycline 7 μg: **** *p* < 0.0001; *** *p* < 0.001.

Using the inhibition halo technique, we evaluated the antimicrobial activity of the EOs of both *C. medica* cultivars. Among the Gram-positive bacteria, *Bacillus cereus* 4384 and *Staphylococcus aureus* were the more sensitive bacterial strains to *C. medica* cv. ‘liscia’ EO (Figure 1).

**Figure 2:**
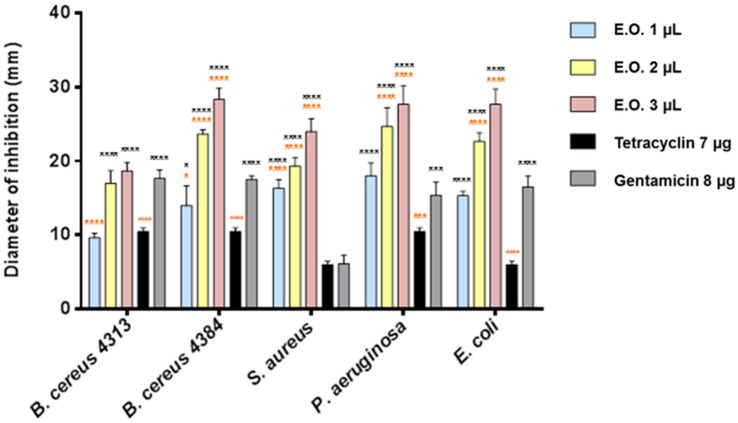
Antibacterial activity of EO of *Citrus medica* cv. ‘rugosa’ and of the reference compounds, gentamicin and tetracycline. Results are the mean of three experiments. Dunnett’s test vs. gentamicin 8 μg: **** *p* < 0.0001; *** *p* < 0.001; * *p* < 0.05; Dunnett’s test vs. tetracycline 7 μg: **** *p* < 0.0001; *** *p* < 0.001; * *p* < 0.05.

Low concentrations of *C. medica* cv. ‘rugosa’ and cv. ‘liscia’ EOs showed strong effect on all tester strains (inhibition halos ranging between 8.5 and 10 mm, Figure 1 and Figure 2). The effect was higher also respect to the antibiotics used as a control. *B. cereus* 4384 resulted in the most sensitive strain; *S. aureus* was the most sensitive at the lowest amount of *Citrus medica* cv. ‘liscia’. In any case, *C. medica* cv. ‘rugosa’ EO showed a greater inhibitory power than *C. medica* cv. ‘liscia’ EO on all bacterial strains and already at low concentrations (Figure 2). Three microliters of the two EOs seemed to give the most effective antimicrobial activity. At this concentration, the behavior exhibited by the pathogen strains was different in terms of sensitivity. Certainly, *C. medica* cv. ‘rugosa’ EO was much more effective than the EO obtained from *C. medica* cv. ‘liscia’. *P. aeruginosa* and *E. coli* were the most sensitive strains to this last EO, with inhibition halos not inferior to 27 mm. A strong activity was also exhibited by *C. medica* cv. ‘rugosa’ EO against *S. aureus*. *C. medica* cv. ‘liscia’ EO was effective, although in not such a marketable mode against the strains, with inhibition halos never superior to 22 mm. The two strains of *B. cereus* used in the experiments (4313 and 4384) reflected, in some way, the different behavior exhibited by the microorganisms used as tester. Both strains were sensitive to the two EOs. *B. cereus* 4313 showed the same behavior, giving similar inhibition halo; on the contrary, *B. cereus* 4384 showed a different behavior, resulting more sensitive to the *C. medica* cv. ‘rugosa’ EO respect to the EO obtained from *C. medica* cv. ‘liscia’ (Δ = 19%). Minimum inhibitory concentration (MIC) values reported a remarkable action of the two EOs. *C. medica* cv. ‘liscia’ EO (MIC = 0.1 μL/mL, Table 2) was more effective than that of *C. medica* cv. ‘rugosa’ (MIC ranging between 0.1 and 0.8 μL/mL), except than against *B. cereus* 4384 (MIC = 0.1 μL/mL) and much more effective against the antibiotic gentamycin. In all cases, the results of the MIC assay indicated that the antimicrobial activity of the two EOs was much more effective than their two main components, limonene and camphene.

### 2.3. Cytotoxicity of Limonene, C. medica cv. ‘liscia’ and C. medica cv. ‘rugosa’ Essential Oils

Limonene, *C. medica* cv. ‘liscia’, and *C. medica* cv. ‘rugosa’ EOs were evaluated for their ability to inhibit the growth of human neuroblastoma cell line (SH-SY5Y). The EOs and limonene revealed different cytotoxic activities. Limonene and *C. medica* cv. ‘rugosa’ EOs showed an IC_50_ > 2000 μg/mL, instead *C. medica* cv. ‘rugosa’ EO showed an IC_50_ of 718.2 μg/mL.

**Figure 3:**
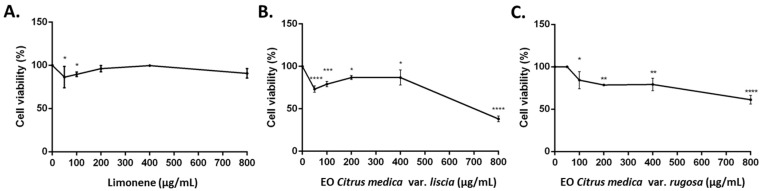
Percentage of cell viability after 3-(4,5-dimethylthiazol-2-yl)-2,5-diphenyl tetrazolium bromide (MTT) assay. Cells were treated with different concentrations (50–800 μg/mL) of limonene (**A**); *C. medica* cv. ‘liscia’ (**B**) and *C. medica* cv. ‘rugosa’ EOs, for 24 h and solvent (DMSO, 0.1%) alone. Data are the mean ± SD of three experiments * *p* < 0.05, ** *p* < 0.01, *** *p* < 0.001, **** *p* < 0.0001 vs. DMSO.

Treatment of SH-SY5Y neuroblastoma cells with 800 μg/mL of limonene for 24 h resulted in a low cytotoxic activity. However, treatment with 800 μg/mL of *C. medica* cv. ‘liscia’ EOs resulted in a stronger cytotoxicity than *C. medica* cv. ‘rugosa’ EO with 38% cell death (Figure 3).

### 2.4. ADCY1: Western Blotting Analysis

**Figure 4:**
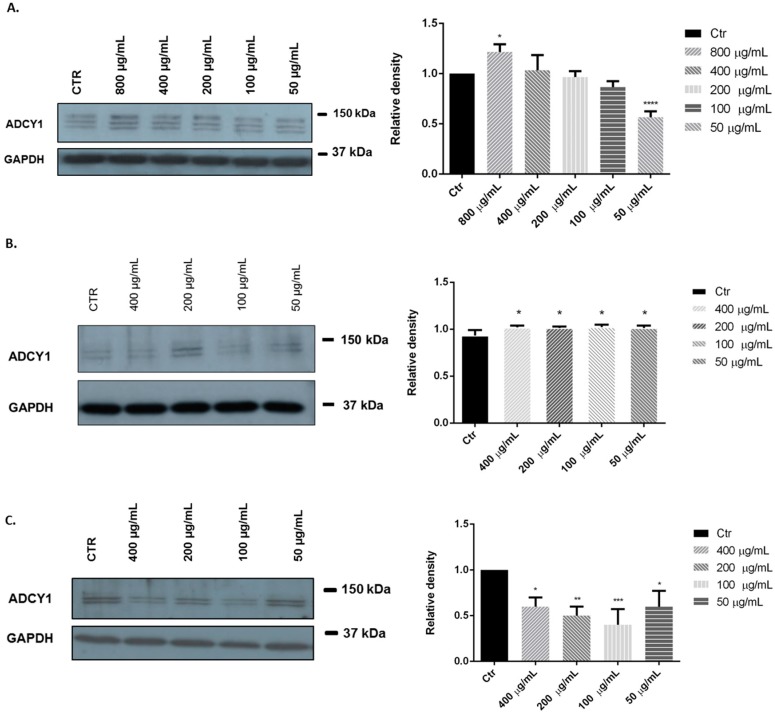
Relative expression levels of the ADCY1 in SH-SY5Y treated with limonene (**A**); *C. medica* cv. ‘liscia’ (**B**); and *C. medica* cv. ‘rugosa (**C**) EOs. Each panel shows a representative Western blot and densitometric analysis of bands in the control and treated groups. Values are the mean ± SD in each group (*n* = 3). * *p* < 0.05, ** *p* < 0.01, *** *p* < 0.001, **** *p* < 0.0001, compared to control (ANOVA followed by Dunnett’s multiple comparison test).

We investigated the effects of limonene, *C. medica* cv. ‘liscia’ and *C. medica* cv. ‘rugosa’ EOs in SH-SY5Y human neuroblastoma cells. Representative Western blots and quantitative densitometric analysis for adenylate cyclase 1 (ADCY1) protein expression in SH-SY5Y following exposure to different concentrations of limonene and EOs are shown in Figure 4. Treatments of SH-SY5Y neuroblastoma cells with 800 and 50 μg/mL of limonene for 24 h significantly influenced ADCY1 expression in different way: high concentrations appear to increase ADCY1 expression, instead low concentrations reduced the ADCY1 expression (Figure 4A). However, treatments with 400, 200, 100, 50 μg/mL of *C. medica* cv. ‘liscia’ and *C. medica* cv. ‘rugosa’ EOs appear to influence significantly ADCY1 expression with an over expression and a down expression of ADCY1, respectively (Figure 4B,C).

## 3. Discussion

There were no relevant quali-quantitative differences in the composition of the two EOs, neither with regard to the percentages of the classes of compounds nor to the main components. In both oils, limonene and camphene are the main components, even if the composition of the EO of *C. medica* cv. ‘rugosa’ is more complex, but many of its constituents are present in very low percentages or even in traces.

The comparison with the available literature concerning the chemical composition of the EO of citron from other countries, showed substantial differences. Limonene percentages found (67.2% and 62.8%) are significantly higher than the values recorded in different parts of the world or even in Italy: South Korea, 52.44% [11]; China, 33.84% [12]; Santa Maria del Cedro (Italy), 59% [1]; and Iran, 56.6% [13]. Other notable differences relate to the content in γ-terpinene exceeding 20% and of α- and β-pinene, reported between 7.73% [12] and 16.3% [13]. Camphene range is 8.5%–10.9% (cv. ‘liscia’ and cv. ‘rugosa’, respectively) in samples studies in this research, but this compound is practically absent or in traces in the oils reported in literature.

The antimicrobial activity of the two EOs was evaluated against different microorganisms: *Bacillus cereus* (DSM 4313 and DSM 4384), an agent of different foodborne illnesses which can be found in raw and processed foods; *Staphylococcus aureus* which plays a significant role in invasive skin disease, and actually representing one of the most significant emerging pathogens; an enterotoxigenic strain of *Escherichia coli*, generally involved in episodes of food poisoning; and *Pseudomonas aeruginosa*, concerned to different urinary, pulmonary, skin, ear and eye infections.

The EO from cv. ’rugosa’ was tested at 1, 2, and 3 mg/mL, because it was more active in antimicrobial tests than the EO from cv. ‘liscia’, tested at 1, 2, 3, 5, and 10 mg/mL.

The modest inhibitory activity exhibited by the two EOs against the Gram-negative strains could be due to the different structures of the cell walls of Gram-positive and Gram-negative bacteria [14]. Our results supported previous studies [15], which reported a different sensitivity shown by the two strains of *B. cereus* against EO of *Hypericum perfoliatum*.

Some authors reported the antimicrobial activity of *C. medica* against different pathogens. Sah and coworkers [16] evaluated the antimicrobial activity of fruit juice and ethanol extract of different plant portion of *Citrus medica* which resulted to be less effective than the EOs of *Citrus medica* cv. liscia and rugosa evaluated in our experiments. Belletti and coworkers evaluated the effects of citron EO on microbial spoilage and growth and survival of pathogenic microorganisms during storage of a fruit based salad, reporting the capability of these substances to prolong the microbial shelf life of these products [17]. Therefore, the values of MIC observed testing limonene and camphene, the two most abundant components present in the two EOs, seemed to corroborate the hypothesis–almost for all pathogens tested–that the activity is due to a synergy among different components, more than to a specific component. In fact, MIC of limonene was always 1 μL, ten-fold higher than that of the two EOs; camphene, generally recognized as good antimicrobial agent [18], was less effective (MIC > 6 μL) except than against *E. coli* (MIC = 0.084 μL). Considering such value and comparing it with the MIC of the two EOs of *Citrus medica*, we could hypothesize a strong influence of camphene on the antimicrobial. On the other hand, these two monoterpenes generally do not exhibit a strong antimicrobial activity [19] per se. Limonene was indicated for its inhibitory activity against the Gram-negative pathogens, *P. aeruginosa* and *E. coli*, but at higher concentrations [20,21,22]. Limonene exists in two enantiomeric forms, with different biological activity. Aggarwal and coworkers have demonstrated that (*S*)-(−)-limonene had less antimicrobial activity than (*R*)-(+)-limonene. In fact, the first failed to inhibit many bacterial strains and the majority of the fungal strains, except *Microsporum gypseum*. In contrast, (*R*)-(+)-limonene was highly active against a wide spectrum of microorganisms [23].

The antimicrobial activity of the two EOs might be due to a global synergy among different singular components, more than to the presence of a specific component.

It is remarkable the stronger antimicrobial activity exhibited by the two EOs. This is still more noteworthy, considering their MIC values. The increasing resistance of microorganisms to conventional chemicals and drugs has prompted scientists to search for novel sources of biocides with broad-spectrum activities. In this concern, the activity of the two EOs studied comforts us towards an ever-greater increase of studies and research aimed at the discovery, exploitation, and potential use of natural substances capable of effectively countering the action of pathogenic microorganisms.

The cytotoxic activity of the two EOs and limonene were evaluated in human neuroblastoma cell line (SH-SY5Y). The IC_50_ values were >700 μg/mL, indicating that the substances were not cytotoxic as judged by the criterion set by the National Cancer Institute that stated that extracts with IC_50_ < 20 μg/mL were considered to be cytotoxic against the treated cells [24].

However, comparing the IC_50_ values, our findings indicated that *C. medica* cv. ‘liscia’ EO is more cytotoxic than *C. medica* cv. ‘rugosa’ EO and limonene. The cytotoxicity can probably be attributed to a synergistic activity of limonene and other minor components. Monajemi and coworkers [13] reported that the EO of *C. limon* with large amounts of limonene (98.4%) was less cytotoxic than *C. medica* with low content of limonene (56.6%) on MCF-7 and HeLa cell lines. Our results agree with previous studies reporting that low concentrations of limonene were ineffective in cell death in SH-SY5Y cells [7,25].

Many plant species with EOs are used as sedatives, hypnotics, and tranquilizers and they may be useful in treating disorders of the central nervous system [26]. Different studies reported a well-established role for adenylyl cyclase in the regulation of synaptic plasticity, memory, and other multiple brain processes. Moreover, cross-talk between the cyclic adenosine-3′,5′-monophosphate (cAMP) signal transduction system and other signaling pathways, such as the extracellular signal-regulated kinase/mitogen-activated protein (ERK/MAP) regulatory system, has been described [27,28]. In this perspective, we evaluated the possible influence of limonene and *C. medica* cv. ‘liscia’ and *C. medica* cv. ‘rugosa’ EOs on ADCY1 expression in SH-SY5Y cells, a human-derived neuroblastoma cell line often used as an in vitro neuronal model [29]. Adenylyl cyclase is an enzyme that regulates the physiological effects of numerous drugs and hormones through the production of cAMP. Clinical and epidemiologic research provided suggestive evidence regarding the association between adenylyl cyclase activity and major depression [30,31]. Moreover, platelet adenylyl cyclase activity might serve as a biological marker for major depression and the therapeutic effect of antidepressants [31,32,33,34].

Our results showed that the higher concentration of limonene (800 μg/mL) appears to increase ADCY1 expression in SH-SY5Y cell and, consequently, the intracellular production of cAMP. Our results agree with results of Watt and coworkers [35] that showed that some volatile substances stimulate cAMP-response element (CRE)-mediated transcription in olfactory sensory neurons through Ca^2+^ activation of the ERK/MAPK/p90rsk (Ribosomal Protein S6 Kinases, 90-kDa) pathway.

*C. medica* cv. ‘liscia’ EO showed similar effects with all concentrations tested, probably because its main component is limonene (67.2%). Limonene is reported to increase cytosolic cAMP concentration and induce activation of protein kinase A and phosphorylation of cAMP-response element-binding protein in Chinese hamster ovary cells transfected with the human adenosine A2A receptor gene [36]. On the contrary, treatment with *C. medica* cv. ‘rugosa’ EO showed an inhibition on ADCY1 expression in SH-SY5Y cell, possibly due to the complex phytochemical composition of this EO. These results showed that the use of both EOs could increase intracellular production of cAMP and have an excitatory effect on the Central Nervous System [26].

## 4. Materials and Methods

### 4.1. Plant Material

Fruits of *Citrus medica* cv. ‘liscia’ and *C. medica* cv. ‘rugosa’ were collected in February 2016 from biological cultivations in the Coast of Amalfi. The plants were identified by Prof. V. De Feo. Voucher specimens (labeled as De Feo/134/2016 for cv. ‘liscia’ and De Feo/133/2016 for cv. ‘rugosa’, respectively) are stored in the Herbarium of the Medical Botany Chair at the University of Salerno.

### 4.2. Isolation of the Essential Oils

One hundred grams of fruit peels of each cultivar were ground in a Waring blender and then subjected to hydrodistillation for 3 h according to the standard procedure described in the European Pharmacopoeia [37]. The oils were solubilized in *n*-hexane, filtered over anhydrous sodium sulphate and stored under N_2_ at 4 °C in the dark, until tested and analyzed.

### 4.3. GC-FID Analysis

Analytical gas chromatography (GC) was carried out on a Perkin-Elmer Sigma-115 gas chromatograph (Perkin Elmer, Waltham, MA, USA) equipped with a flame ionization detector (FID) and a data handling processor. The separation was achieved using a HP-5 MS fused-silica capillary column (30 m × 0.25 mm i.d., 0.25 μm film thickness, Agilent, Roma, Italy). Column temperature: 40 °C, with 5 min initial hold, and then to 270 °C at 2 °C/min, 270 °C (20 min); injection mode, splitless (1 μL of a 1:1000 *n*-hexane solution). Injector and detector temperatures were 250 °C and 290 °C, respectively. Analysis was also run by using a fused silica HP Innowax polyethylene glycol capillary column (50 m × 0.20 mm i.d., 0.25 μm film thickness, Agilent). In both cases, helium was used as the carrier gas (1.0 mL/min).

### 4.4. GC/MS Analysis

Analysis was performed on an Agilent 6850 Ser. II apparatus (Agilent), fitted with a fused silica DB-5 capillary column (30 m × 0.25 mm i.d., 0.33 μm film thickness, Agilent), coupled to an Agilent Mass Selective Detector MSD 5973; ionization energy voltage 70 eV; electron multiplier voltage energy 2000 V. Mass spectra (MS) were scanned in the range 40–500 amu, scan time 5 scans/s. Gas chromatographic conditions were as reported in the previous paragraph; transfer line temperature, 295 °C.

### 4.5. Identification of the Essential Oil Components

Most constituents were identified by GC by comparison of their Kovats retention indices (Ri) (determined relative to the retention times (tR) of *n*-alkanes (C10–C35)), with either those of the literature [38,39,40,41] and mass spectra on both columns or those of authentic compounds available in our laboratories by means of NIST 02 and Wiley 275 libraries [42]. The components’ relative concentrations were obtained by peak area normalization. No response factors were calculated.

### 4.6. Antimicrobial Activity

To study antimicrobial activity a filter paper disc method was used [43]. The bacteria used in this study included *Bacillus cereus* DSM 4313, *Bacillus cereus* 4384, *Staphylococcus aureus* DSM 25693, *Pseudomonas aeruginosa* ATCC 50071, and *Escherichia coli* DSM 8579. Bacteria were purchased from the Deutsche SammLung von Mikroorganismen und Zellkulturen GmbH (DSMZ, Deutsche Sammlung von Mikroorganismen und Zellkulturen, Braunschweig, Germany).

A 100 mg sample of each EO was re-suspended in dimethyl sulfoxide (DMSO) to produce the same concentration of 10 mg/mL, then diluted to be subjected to biological analyses. The strains were incubated in nutrient broth (Oxoid, Milano, Italy) at 37 °C for 18 h. The optical densities of all cultures were adjusted to match a 0.5 McFarland standard of 1 × 10^8^ colony-forming units (cfu)/mL. Sterile filter paper discs (5 mm) were impregnated with 1 and 10 μL of the EOs and placed in Petri dishes. Plates were left for 30 min at room temperature under sterile conditions and then incubated at 37 °C for 24 h, and the inhibition halo around the disc was measured. A disc treated with DMSO alone served as the negative control. Tetracycline (7 μg/disc; Sigma) and gentamycin (8 μg/disc; Sigma) were used as reference drugs. The experiments were performed in triplicate and averaged.

### 4.7. Minimum Inhibitory Concentration

For the MIC of the EOs and their main components, limonene and camphene, a modified version of the resazurin microtiter-plate assay of Sarker and coworkers was used [44]. Briefly, different volumes of the two EOs, prepared as above described, and of the pure compounds limonene (dissolved in DMSO, 1:10 *v*/*v*), and camphene (dissolved in a mix hexane/DMSO 1:10 *v*/*v*), were used and pipetted into the first row of the 96 well plates. To all wells, different volumes of Muller-Hinton broth was added. Two-fold serial dilutions were performed such that each well had 50 μL of the test material in serially descending concentrations. 30 μL of 3.3× strength isosensitised broth and 10 μL of resazurin indicator solution (previously prepared by dissolving 270 mg tablet in 40 mL of sterile distilled water) were added in each well, to reach a final volume/well of 240 μL. Finally, 10 μL of bacterial suspension was added to each well to achieve a concentration of approx. 5 × 10^5^ cfu/mL. Each plate was wrapped loosely with cling film to ensure that bacteria did not become dehydrated. In each plate a set of controls were added: a column with a ciprofloxacin (1 mg/mL in DMSO, Sigma) as positive control; a column with all solutions with the exception of the test compound; a column with all solutions with the exception of the bacterial solution adding 10 μL of nutrient broth instead and a column with DMSO solution as a negative control. The plates were prepared in triplicate, and incubated at 37 °C for 24 h. The growth was indicated by color changes (visually assessed) from blue/dark purple to pink (or colorless). The lowest concentration at which color change occurred indicated the MIC value.

### 4.8. Cell Cultures

Human neuroblastoma (SH-SY5Y) cancer cells were cultured in Roswell Park Memorial Institute Medium (RPMI) medium supplemented with 1% l-glutamine, 10% heat-inactivated fetal bovine serum (FBS), 1% penicillin/streptomycin (all from Sigma-Aldrich, St. Louis, MO, USA) at 37 °C in an atmosphere of 95% O_2_ and 5% CO_2_.

### 4.9. MTT Bioassay

Human neuroblastoma cancer cells (SH-SY5Y) were plated (5 × 10^3^) in 96-well culture plates in 150 μL of culture medium and incubated at 37 °C in humidified 5% CO_2_. The day after, a 150 μL aliquot of serial dilutions of limonene and EOs (800–50 μg/mL) was added to the cells and incubated for 24 h. DMSO alone was used as control. Cell viability was assessed through 3-(4,5-dimethylthiazol-2-yl)-2,5-diphenyl tetrazolium bromide (MTT) assay. Briefly, 30 μL of MTT (5 mg/mL) was added and the cells incubated for additional 3 h. Thereafter, cells were lysed and the dark blue crystals solubilized with 30 μL of a solution containing 50% *v*/*v* *N*,*N*-dimethylformamide and 20% *w*/*v* SDS with an adjusted pH of 4.5. The optical density (OD) of each well was measured with a microplate spectrophotometer (Thermo Scientific Multiskan GO, Monza, Italy) equipped with a 520 nm filter. Cell viability in response to treatment was calculated as a percentage of control cells treated with DMSO at the final concentration (0.1%): viable cells = (100 × OD treated cells)/OD control cells [45].

### 4.10. Extraction Proteins and Western Blotting

Cells were treated with different concentrations (800–100 μg/mL) of limonene and EOs. After 24 h, cells were collected and lysed using LaemmLi buffer to extract total proteins. For Western blot analysis, an aliquot of total protein was run on 8% SDS-PAGE gel and transferred to nitrocellulose. Nitrocellulose blots were blocked with 10% nonfat dry milk in Tris buffer saline 0.1% Tween-20 over night at 4 °C and incubated with primary anti-ADCY1 (Santa Cruz Biotechnology, Santa Cruz, CA, USA) for 3 h at room temperature. Immunoreactivity was detected by sequential incubation with horseradish peroxidase-conjugated secondary antibody (Amersham Biosciences, Pittsburgh, PA, USA) and enhanced chemiluminescence reagents (ImmunoCruz, Santa Cruz Biotechnology) [46].

### 4.11. Statistical Analysis

All experiments were carried out in triplicate. Data of each experiment were statistically analyzed using GraphPad Prism 6.0 software (GraphPad Software Inc., San Diego, CA, USA) followed by comparison of means (two-way ANOVA) using Dunnett’s multiple comparisons test, at the significance level of *p* < 0.05.

## 5. Conclusions

We characterized the chemical composition of the EOs of two *C. medica* cultivars, characteristics of Amalfi Coast. These oils present a new composition, with high presence of limonene, which seems a ’fingerprint’ of these oils. The EOs showed higher antimicrobial activity at the tested doses. The EOs showed higher antimicrobial activity than antibiotics used as control. The presence of different components present in the two oils, more than the high content of limonene and camphene might be responsible for this EOs activity. This finding led us to hypothesize the possible use of these oils as antimicrobials and confirm their traditional uses as food preserving agents.

The alterations in ADCY1 expression suggested a major role for limonene in effects on the central nervous system. The precise mechanism of the biochemical effects of *Citrus medica* EOs is unclear; however, ADCY1 pathway might be involved. Moreover, the chemical and biological study of these EOs contribute to the knowledge and promotion of two cultivars belonging to the biodiversity of South Italy.

## Figures and Tables

**Table 1:** Chemical composition of the essential oils (EOs) isolated from the peels of *C. medica* cv. ‘liscia’ (CL) and *C. medica* cv. ‘rugosa’ (CR) grown in Amalfi Coast.

No.	Compound	LRI ^a^	LRI ^b^	CL	CR	Identification ^c^
1	α-Thujene	915	930	-	0.1	1, 2
2	α-Pinene	921	939	0.8	1.2	1, 2
3	α-Fenchene	934	952	0.1	0.1	1, 2
4	Camphene	964	954	8.5	10.9	1, 2, 3
5	β-Pinene	980	979	1.4	1.7	1, 2
6	α-Phellandrene	991	1002	0.5	0.6	1, 2
7	δ-2-Carene	1004	1002	0.1	0.3	1, 2
8	*p*-Cymene	1012	1024	-	1.0	1, 2
9	Limonene	1022	1029	67.2	62.8	1, 2
10	(*Z*)-β-Ocimene	1028	1037	Tr	0.1	1, 2
11	(*E*)-β-Ocimene	1038	1050	0.1	0.3	1, 2, 3
12	γ-Terpinene	1047	1059	0.3	0.7	1, 2
13	Linalool oxide furanoid	1064	1072	0.3	Tr	1, 2
14	*trans*-Linalool oxide		1086	0.1	-	1, 2
15	Terpinolene	1077	1088	0.1	0.3	1, 2
16	Linalool	1091	1096	0.3	1.3	1, 2
17	α-Pinene oxide	1095	1099	Tr	0.1	1, 2
18	1,3,8-*p*-Menthatriene	1100	1110	-	Tr	1, 2
19	Perillene	1103	1103	Tr	Tr	1, 2
20	*trans*-Thujone	1106	1114	Tr	0.1	1, 2, 3
21	Dehydro sabina ketone	1111	1120	0.1	0.1	1, 2
22	*allo*-Ocimene	1119	1132	Tr	0.1	1, 2
23	*cis-p*-Mentha-2,8-dien-1-ol	1126	1137	-	Tr	1, 2
24	*cis*-Limonene oxide	1133	1136	Tr	0.5	1, 2
25	*trans*-Limonene oxide	1140	1142	-	Tr	1, 2, 3
26	Isopulegol	1144	1149	Tr	0.1	1, 2
27	*neo allo*-Ocimene	1152	1144	-	Tr	1, 2
28	Citronellal	1155	1153	Tr	0.2	1, 2
29	*neo iso*-Isopulegol	1167	1171	0.8	0.7	1, 2
30	Isoborneol	1163	1160	-	Tr	1, 2, 3
31	α-Terpineol	1180	1188	0.7	0.6	1, 2
32	Hexyl butanoate	1183	1192	-	Tr	1, 2
33	Dihydrocarveol	1185	1193	Tr	Tr	1, 2
34	Methyl chavicol	1190	1196	-	Tr	1, 2
35	*trans*-4-Caranone	1195	1196	0.3	0.1	1, 2, 3
36	Decenal	1198	1196	-	Tr	1, 2
37	2-Decanol	1202	1199	0.3	0.1	1, 2
38	*cis*-4-Caranone	1209	1200	0.1	0.3	1, 2
39	*endo*-Fenchyl acetate	1219	1220	0.9	0.4	1, 2
40	Thymol methyl-ether	1223	1235	-	Tr	1, 2
41	Neral	1231	1238	0.1	0.5	1, 2
42	Geraniol	1246	1252	0.9	0.7	1, 2, 3
43	Geranial	1261	1267	0.1	0.7	1, 2
44	*n*-Decanol	1263	1269	0.3	-	1, 2
45	*trans*-Carvone oxide	1276	1276	0.1	0.1	1, 2, 3
46	Thymol	1283	1290	-	0.4	1, 2, 3
47	*p*-Cymene-7-ol	1292	1290	-	Tr	1, 2
48	Undecen-10-en-1-al	1296	1299	0.1	Tr	1, 2
49	*n*-Nonanyl acetate	1301	1312	0.1	Tr	1, 2
50	Citronellic acid	1314	1313	Tr	Tr	1, 2
51	δ-Elemene	1326	1338	0.4	0.2	1, 2, 3
52	α-Terpinyl acetate	1339	1349	Tr	0.1	1, 2, 3
53	Citronellyl acetate	1343	1352	0.1	0.1	1, 2, 3
54	Eugenol	1348	1359	-	Tr	1, 2
55	Neryl acetate	1354	1361	0.7	0.6	1, 2, 3
56	α-Ylangene	1364	1375	Tr	Tr	1, 2, 3
57	α-Copaene	1368	1376	-	Tr	1, 2, 3
58	Geranyl acetate	1373	1381	0.9	0,5	1, 2, 3
59	β-Patchoulene	1380	1381	0.1	0.1	1, 2
60	Methyl eugenol	1396	1403	0.1	0.1	1, 2, 3
61	Italicene	1399	1405	0.1	Tr	1, 2
62	Sesquithujiene	1403	1405	0.1	Tr	1, 2
63	Longifolene	1407	1407	0.5	0.6	1, 2, 3
64	β-Duprezianene	1417	1422	0.1	0.1	1, 2
65	γ-Elemene	1422	1436	0.1	0.1	1, 2, 3
66	α-*trans*-Bergamotene	1424	1434	0.5	0.4	1, 2
67	α-Guaiene	1432	1439	Tr	Tr	1, 2, 3
68	Aromadendrene	1441	1441	0.1	0.1	1, 2, 3
69	(*Z*)-β-Farnesene	1445	1442	0.1	0.1	1, 2, 3
70	(*E*)-β-Farnesene	1449	1456	Tr	Tr	1, 2
71	*cis*-Cadin-1(6),4-diene	1457	1463	-	Tr	1, 2
72	9-*epi*-(*E*)-Caryophyllene	1469	1466	Tr	0.1	1, 2, 3
73	β-Acoradiene	1473	1470	Tr	Tr	1, 2
74	γ-Gurjenene	1478	1477	Tr	-	1, 2
75	α-Amorphene	1482	1484	0.1	Tr	1, 2, 3
76	Aristolochene	1486	1488	Tr	Tr	1, 2
77	β-Selinene	1490	1490	0.1	0.1	1, 2
78	α-Selinene	1496	1498	1.0	0.6	1, 2
79	α-Cuprenene	1502	1505	0.1	Tr	1, 2
80	δ-Amorphene	1511	1512	-	0.1	1, 2
81	δ-Cadinene	1523	1523	0.1	-	1, 2
82	(*Z*)-Nerolidol	1526	1532	Tr	-	1, 2
83	γ-Cuprenene	1530	1533	Tr	Tr	1, 2
84	(*E*)-Nerolidol	1552	1563	0.3	Tr	1, 2
85	Caryophyllene oxide	1572	1583	-	0.1	1, 2, 3
86	Globulol	1580	1590	Tr	Tr	1, 2
87	β-Oplopenone	1597	1607	Tr	Tr	1, 2
88	Guaiol	1599	1600	0.1	Tr	1, 2
89	1-*epi*-Cubenol	1618	1628	Tr	Tr	1, 2
90	Eremoligenol	1629	1631	Tr	-	1, 2
91	α-Muurolol	1631	1646	Tr	-	1, 2, 3
92	*epi*-α-Muurolol	1644	1642	0.1	0.1	1, 2
93	Pogostol	1647	1653	0.3	Tr	1, 2
94	Cedranol	1658	1673	0.1	0,1	1, 2
95	α-Bisabolol	1674	1685	0.1	-	1, 2, 3
96	Eudesm-7(11)-en-4-ol	1682	1700	Tr	0.5	1, 2
97	*Z*-α-trans-Bergamotol	1688	1690	0.1	-	1, 2
98	Nootkatol	1703	1715	0.3	-	1, 2
99	(2*Z*,6*E*)-Farnesol	1711	1723	Tr	-	1, 2
100	Oplopanone	1717	1740	Tr	-	1, 2
	Monoterpene hydrocarbons			79.1	80.2	
	Oxygenated monoterpenes			4.8	6.9	
	Sesquiterpene hydrocarbons			4.2	3.2	
	Oxygenated sesquiterpenes			2.5	1.6	
	Non terpenes			0.8	0.1	
	Total			91.4	92.0	

^a^ Linear retention index on a HP-5MS column; ^b^ Linear retention index on a HP Innowax column; ^c^ Identification method: 1 = linear retention index; 2 = identification based on the comparison of mass spectra; 3 = Co-Gas chromatography retention time identical to authentic compounds; -: not detected; Tr = trace (<0.1%).

**Table 2:** Minimal inhibitory concentration (MIC, μL) of the EOs of *Citrus medica* cv. ‘liscia’ and cv. ‘rugosa’ and of their main components, limonene and camphene.

Microorganism	MIC (μL)			
	*C. medica* cv. ‘Liscia’	*C. medica* cv. ‘Rugosa’	Limonene	Camphene
*Bacillus cereus* 4313	0.1 μL	0.5 μL	1 μL	>6 μL
*Bacillus cereus* 4384	0.1 μL	0.1 μL	1 μL	>8 μL
*Escherichia coli*	0.1 μL	0.2 μL	1μL	0.084 μL
*Pseudomonas aeruginosa*	0.1 μL	0.8 μL	1 μL	>10 μL
*Staphylococcus aureus*	0.1 μL	0.8 μL	1 μL	>10 μL

## Abbreviations

The following abbreviations were used in this manuscript:

ADCY1	Adenylate Cyclase 1
cAMP	Cyclic adenosine-3′,5′-monophosphate
EO	Essential oil
ERK	Extracellular signal-regulated kinase
MAP	Mitogen-activated protein
MTT	3-(4,5-dimethylthiazol-2-yl)-2,5-diphenyl tetrazolium bromide
p90rsk	Ribosomal Protein S6 Kinases, 90-kDa
RPMI	Roswell Park Memorial Institute Medium
OD	Optical Density
